# Dr. Geo. A. Otis

**Published:** 1881-05

**Authors:** 


OBITUARY.—Died at Washington, on the 23d of February, aged fifty years, George Alexander Otis, M.D., Surgeon U. S. A. Dr. Otis, who is widely known as an eminent writer on military surgery, and as the compiler of the surgical volumes of the Medical and Surgical History of the War, was born at Boston, Massachusetts, November 12, 1830; he graduated in the Arts at Princeton College, and in Medicine at the University of Pennsylvania, in 1850. He then visited Europe and prosecuted his studies in London and Paris, and, returning to this country, he began the practice of his profession at Springfield, Massachusetts. He entered the army as surgeon of the 27th Massachusetts' Volunteers, in September, 1861, and after the close of the war, he entered the medical corps of the regular army.—*Medical News*, April.

